# Major QTL for Seven Yield-Related Traits in Common Wheat (Triticum aestivum L.)

**DOI:** 10.3389/fgene.2020.01012

**Published:** 2020-08-28

**Authors:** Jingjing Jin, Dan Liu, Yongzhi Qi, Jun Ma, Wenchao Zhen

**Affiliations:** ^1^College of Plant Protection, Hebei Agricultural University, Baoding, China; ^2^State Key Laboratory of North China Crop Improvement and Regulation, Baoding, China; ^3^Neijiang Academy of Agricultural Sciences, Neijiang, China; ^4^School of Life Sciences and Engineering, Southwest University of Science and Technology, Mianyang, China; ^5^College of Agronomy and Biotechnology, China Agricultural University, Beijing, China; ^6^College of Agronomy, Hebei Agricultural University, Baoding, China

**Keywords:** wheat, recombinant inbred line, yield-related traits, quantitative trait loci, candidate genes

## Abstract

Flag leaves, plant height (PH), and spike-related traits are key determinants contributing to yield potential in wheat. In this study, we developed a recombinant inbred line (RIL) population with 94 lines derived from the cross between ‘AS985472’ and ‘Sumai 3.’ A genetic map spanned 3553.69 cM in length were constructed using 1978 DArT markers. Severn traits including flag leaf width (FLW), flag leaf length (FLL), PH, anthesis date (AD), spike length (SL), spikelet number spike (SNS), and spike density (SD) were evaluated against this RIL population under three different environments. Combined phenotypic data and genetic map, we identified quantitative trait loci (QTL) for each trait. A total of four major and stably expressed QTLs for FLW, PH, and SD were detected on chromosomes 2D and 4B. Of them, the major QTLs individually explained 10.10 – 30.68% of the phenotypic variation. QTLs with pleiotropic effects were identified on chromosomes 4A and 6D as well. Furthermore, the genetic relationships between seven yield-related traits were detected and discussed. A few genes related to leaf growth and development at the interval of a major locus for FLW on chromosome 2D were predicated. Overall, the present study provided useful information for understanding the genetic basis of yield-related traits and will be useful for marker-assisted selection in wheat breeding.

## Introduction

Ninety-five percent of the energy in nature comes from photosynthesis(Zhai et al., 2002) and the leaves are the major photosynthetic organ in plants. Flag leaf, the first leaf under the spike of wheat (Triticum aestivum L.), contributes to photosynthesis and provides water and nutrients to the spikes for grain filling (Yang et al., 2016). Other agronomic traits like anthesis date (AD), spike length (SL), spikelet number per spike (SNS), and spike density (SD) are also key determinants of the plant architecture and yield potential. It is known that grain yield is closely correlated with AD (Woodruff and Tonks, 1983), plant height (PH) (Hedden, 2003), SL (Liu et al., 2018b), SNS (Hai et al., 2008), and SD (Li et al., 2016). Thus, a comprehensive understanding of the genetic mechanism for flag leaf width (FLW), flag leaf length (FLL), PH and spike-related traits is critical for increasing grain yield.

Agronomic traits are usually controlled by multiple genes and numerous quantitative trait loci (QTL) for them have been reported on A, B, and D genomes in wheat. For instance, in hexaploid wheat, major QTLs for FLL, FLW, flag leaf area (FLA), the ratio of length/width of flag leaf (FLR), flag leaf angle (FLANG), fag leaf opening angle (FLOA) and fag leaf bend angle (FLBA) were mapped to chromosomes 2D, 5B, 4B (Ma et al., 2020). Liu et al. (2018c) detected QTLs for FLL, FLW, FLA and FLANG on chromosomes 1B, 2B, 3A, 3D, 4B, 5A, 6B, 7B, and 7D using a recombinant inbred line (RIL) population derived from ‘ND3331’ and ‘Zang1817.’ Hu et al. (2020) identified 161 QTLs for yield-related traits including grain yield per plant (GYP), spike number per plant (SN), kernel number per spike (KPS), SL, SNS, FLL, FLW, FLA, PH, AD and heading date (HD) on 21 chromosomes except 2D, 3D, and 6D. Although studies on traits related to flag leaf and spike have made great progress, there are still many novel loci that can be excavated and utilized from different germplasm resources.

Significant correlations between agronomic traits of wheat were observed in numerous studies. For example, a study of phenotypic correlations showed that SNS was significantly and positively correlated with SL, AD, and KPS (Ma et al., 2019a). Furthermore, QTLs or genes with pleiotropic effects on agronomic traits in wheat have been previously verified. For example, QTLs with pleiotropic effects to SN, SL, and KPS were identified on chromosomes 1B, 4B, and 5A (Deng et al., 2011). Similarly, Ma et al. (2020) detected two pleiotropic QTLs associated with FLL and FLR on chromosomes 5B, two pleiotropic QTLs for FLOA and FLBA on chromosomes 2D, and three pleiotropic QTLs for FLL, FLW and FLA on chromosomes 2D, and they shared the same or overlapped physical intervals on ‘Chinese Spring’ (CS) genomes. Additionally, these pleiotropic QTLs exhibited significant associations in Pearson correlation analysis. Thus, pleiotropic or linked loci could benefit improving breeding efficiency for multiple elite traits.

The present study focused on detecting QTLs controlling flag leaf traits including FLL and FLW, and spike-related traits including SNS, SL and SD, and AD and PH in a RIL population developed from the cross between ‘AS985472’ and ‘Sumai 3,’ and evaluating their genetic correlations. This study will provide valuable information to understand the genetic basis of yield-related traits and help to accelerate molecular assisted breeding in wheat.

## Materials and Methods

### Plant Materials

A total of 94 F_8_ RILs generated from the cross of ‘AS985472’/‘Sumai 3’ (AS) were used in the present study. ‘AS985472’ is an advance wheat line; ‘Sumai 3’ is an excellent germplasm resource with high resistance to Fusarium head blight (Xie et al., 2007).

### Field Trials and Phenotypic Evaluation

From 2017 to 2019, 94 AS RILs and the two parents were planted at Chongzhou (CZ, 103° 38′ E, 30° 32′ N) of Sichuan Province with a random block design. Each line was in a single 1.5-m row with 30-cm apart between rows, and 15 seeds were planted in each row with 10-cm space between individuals.

Anthesis date (d) was calculated from the sowing date to date when more than 50% of the plants of a line flowered. After anthesis, FLW (cm) was determined by the widest section of the flag leaf, FLL (cm) was measured as the length from the base to the top of the flag leaf. The measurements of PH (cm), SL (cm), SNS and SD were carried out as described by Ma et al. (2019a). PH was obtained by measuring the height from the base to the top of the main spike excluding the awns. SL was the length of the main spike of an individual plant (excluding awns). SNS was determined by the number of spikelets of the spike for the main tiller and SD was SNS divided by SL.

### Statistical Analysis and QTLs Detection

Data obtained from 2017 to 2019 in CZ were subjected to combined analysis. The mean values and the Student’s t-test (P < 0.05) of the parental lines were calculated. For each RIL, the maximum and minimum values, mean values and standard deviation were analyzed using the SPSS Statistic 25 (IBM SPSS, Armonk, NY, United States).

To estimate random effects in statistics, the best linear unbiased prediction (BLUP) for seven yield-related traits in different environments were calculated using SAS version 8.0 (SAS Institute, Cary, NC, United States). The BLUP for the phenotypic values were calculated according to the model: Y_i_ = X_i_f+ a_i_ + e_i_, where f = a vector of fixed effects, Xi = an incidence vector, e_i_ = the environmental deviation, and a_i_ = the phenotypic value (Goddard, 1992). The broad-sense heritability (H^2^) was estimated using the following formula: H^2^**=** V_G_/(V_G_ + V_GE_/r + V_E_). Where V_G_ = genetic variance, V_GE_ = genotype × environment variance, r = the number of replicates, and V_E_ = environ mental variance (Smith et al., 1998).

The genetic linkage map was constructed according to a previous study (Liu et al., submitted). To retain high confidence markers, minor allele frequency (<0.3) of the markers were excluded using the BIN function of Icimapping 4.1 and the linkage group for AS population was integrated using the software Joinmap 4.0 according to Liu et al. (2018a). The linkage map was 3553.69 cM in length containing 31 linkage groups and 1978 DArT markers (Liu et al., submitted). Then, the putative QTLs were detected with a minimum LOD (log-of-odds) value of 2.5 by the BIP (Biparental Populations) module (Lin et al., 1996) and ICIM (inclusive composite interval mapping) method from Icimapping 4.1. Among the detected QTLs, those with >10% of phenotypic variation and could be detected in at least two tested environments as well as the BLUP dataset were treated as major stable QTLs and those with a common flanking marker were considered as a single QTL.

### Comparison of QTLs Related to FLW on 2D

The physical positions of the major QTLs on the genome assembly of T. aestivum cv. Chinese Spring or CS (IWGSC RefSeq v1.0)^1^ (The International Wheat Genome Sequencing Consortium, 2018), Ae. tauschii (Aet V4.0^2^) (Luo et al., 2017), and T. turgidum^3^ (Avni et al., 2017) and the analysis of candidate genes within the interval between the flanking markers on CS genome were obtained according to previous studies (Ma et al., 2020). Additionally, the physical positions of the major loci detected in the present study were compared with the reported QTLs or genes.

## Results

### Phenotypic Performance of the ES RILs

**FIGURE 1:**
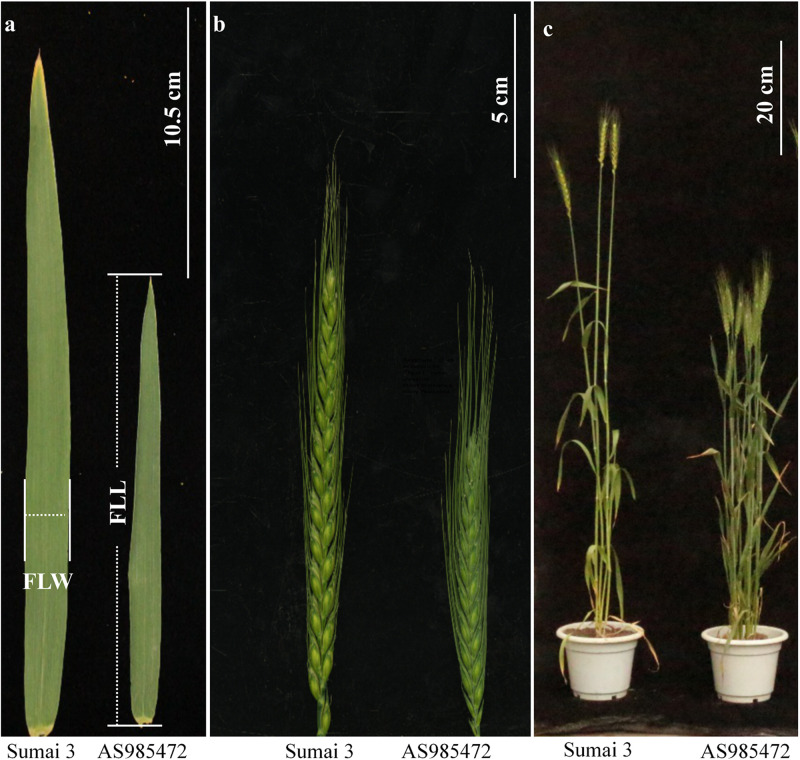
Morphology of the flag leaf **(a)**, spike **(b)**, and plant architecture **(c)** of ‘Sumai 3’ and ‘AS985472’. (Scale bar = 10.5, 5, and 20 cm, respectively).

**FIGURE 2:**
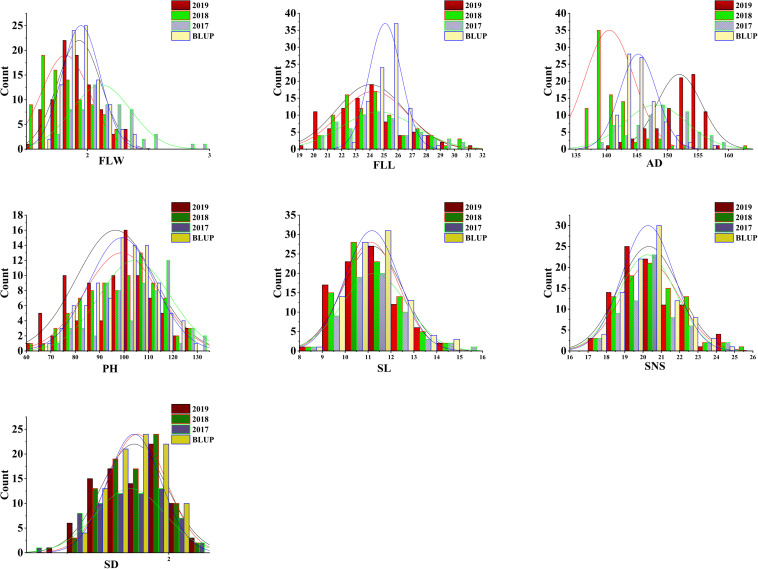
Phenotypic distribution of wheat flag leaf width (FLW), flag leaf length (FLL), anthesis date (AD), plant height (PH), spike length (SL), spikelet number per spike (SNS) and spike density (SD) in the ‘AS985472’/‘Sumai 3’ or AS recombinant inbred population.

The analyses of phenotypic variation showed that significant differences existed between ‘AS985472’ and ‘Sumai 3’ (P < 0.05, Table 1 and Figure 1). ‘Sumai 3’ had wider FLW, longer FLL, higher PH, more SNS, longer spike but lower SD, later AD than ‘AS985472.’ In addition, the continuous distributions with ranges from 1.52 to 2.90 cm in FLW, 19.41 to 31.05 cm for FLL, 136 to 162 d for AD, 61.67 to 131.17 cm for PH, 8.2 to 15.63 cm for SL, 17 to 25 for SNS and 1.37 to 2.15 for SD (Table 1) and transgressive segregation across all environments as well as in the BLUP datasets (Figure 2) indicated that the RILs were suitable for QTL analysis. The estimated H^2^ of FLW, FLL, AD, PH, SL, SNS, and SD for ES RILs were ranged from 0.75 to 0.97, SL had the highest H^2^ (0.97), followed by PH (0.95), and SD had the lowest H^2^ (0.75), implicating that these traits were mainly controlled by genetic factors.

**TABLE 1:** Phenotypic variation and heritability (H^2^) for seven yield-related traits of the ‘AS985472’/ ‘Sumai 3’ (AS) population in different environments.

**Trait**	**Environment**	**Parents**		**AS985472/Sumai 3**			
		**AS985472**	**Sumai 3**	**Min-Max**	**Mean**	**STD**	**H^2^**
FLW (cm)	2019CZ	1.85	2.03*	1.59–2.38	1.93	0.18	
	2018CZ	1.70	1.88*	1.52–2.23	1.82	0.20	
	2017CZ	2.08	2.30**	1.70–2.90	2.11	0.24	
	BLUP	1.94	2.00	1.68–2.40	1.95	0.16	0.88
FLL (cm)	2019CZ	20.90	25.57**	19.41–31.05	24.05	2.43	
	2018CZ	22.89	30.25**	20.24–30.41	24.26	2.43	
	2017CZ	25.53	29.70**	20.00–30.40	24.62	2.63	
	BLUP	23.95	26.76	22.73–28.75	25.09	1.14	0.94
AD (d)	2019CZ	154.00	144.00	141.00–158.00	151.94	3.67	
	2018CZ	142.00	136.00	136.00–162.00	140.49	3.99	
	2017CZ	153.00	141.00	139.00–158.00	148.05	5.10	
	BLUP	149.11	141.76	140.27–157.83	145.21	3.02	0.75
PH (cm)	2019CZ	88.70	113.80**	62.10–129.90	96.46	15.94	
	2018CZ	85.33	109.67**	61.67–128.57	99.00	15.02	
	2017CZ	80.17	118.00**	70.33–131.17	104.34	14.56	
	BLUP	85.43	113.11	68.10–126.73	99.12	13.46	0.95
SL (cm)	2019CZ	9.67	13.99**	8.69–14.28	11.19	1.29	
	2018CZ	9.97	13.42**	8.40–14.60	11.13	1.22	
	2017CZ	10.77	15.63**	8.20–15.63	11.28	1.41	
	BLUP	10.16	14.26	8.51–14.44	11.17	1.18	0.97
SNS	2019CZ	19.20	21.40**	17.25–24.75	20.32	1.65	
	2018CZ	19.33	22.00*	17.00–25.00	20.39	1.61	
	2017CZ	19.67	22.00*	17.33–24.33	20.24	1.56	
	BLUP	19.44	21.51	17.42–24.49	20.27	1.43	0.92
SD	2019CZ	2.00**	1.54	1.49–2.15	1.83	0.16	
	2018CZ	1.94*	1.56	1.51–2.14	1.84	0.14	
	2017CZ	1.83**	1.37	1.37–2.15	1.81	0.17	
	BLUP	1.92	1.52	1.54–2.08	1.83	0.13	0.93

FLW, flag leaf width; FLL, flag leaf length; AD, anthesis date; PH, plant height; SL, spike length; SNS, spikelet number spike; SD, spike density; STD, standard deviation; H^2^, the broad-sense heritability; BLUP, best linear unbiased prediction;**significance level at P = 0.01; *significance level at P = 0.05; CZ, Chongzhou.

### Relationships Among Seven Agronomic Traits

In the three environments as well as the BLUP dataset, for FLW, AD, FLL, PH, SL, SNS, and SD, significant correlations with coefficients ranging from 0.444 to 0.978 were detected (P < 0.05; Table 2). Correlation coefficients between seven agronomic traits using the BLUP dataset were presented in Table 3. FLW was significantly correlated to SNS (r = 0.230, P < 0.05), whereas there were no significant differences between FLW and other yield-related traits. For AD, positive and significant relationships (0.269 ≤ r ≤ 0.446, P < 0.01) between AD and FLL, SL and SNS were observed. FLL was significantly associated with AD, SL, and SNS (0.269 ≤ r ≤ 0.455, P < 0.01). PH was positively and significantly associated with SL and SNS (0.237 ≤ r ≤ 0.446, P < 0.05). Regarding SL, positive and significant correlations between SL and AD, FLL, PH and SNS were detected (0.446 ≤ r ≤ 0.668, P < 0.01). SNS was positively and significantly correlated with AD, FLW, FLL, PH, and SL (0.230 ≤ r ≤ 0.668, P < 0.05). For SD, SD was negatively and significantly related to AD, FLL, PH and SL (−0.724 ≤ r ≤ −0.229, P < 0.05).

**TABLE 2:** Phenotypic correlations of seven yield-related traits in different environments.

**Trait**	**Environment**	**2019CZ**	**2018CZ**	**2017CZ**	**BLUP**
FLW (cm)	2019CZ	1			
	2018CZ	0.744**	1		
	2017CZ	0.782**	0.676**	1	
	BLUP	0.921**	0.895**	0.923**	1
FLL (cm)	2019CZ	1			
	2018CZ	0.910**	1		
	2017CZ	0.897**	0.954**	1	
	BLUP	0.810**	0.819**	0.780**	1
AD (d)	2019CZ	1			
	2018CZ	0.444**	1		
	2017CZ	0.633**	0.654**	1	
	BLUP	0.659**	0.891**	0.950**	1
PH (cm)	2019CZ	1			
	2018CZ	0.852**	1		
	2017CZ	0.863**	0.887**	1	
	BLUP	0.956**	0.957**	0.958**	1
SL (cm)	2019CZ	1			
	2018CZ	0.921**	1		
	2017CZ	0.926**	0.934**	1	
	BLUP	0.973**	0.975**	0.978**	1
SNS	2019CZ	1			
	2018CZ	0.869**	1		
	2017CZ	0.863**	0.874**	1	
	BLUP	0.950**	0.960**	0.960**	1
SD	2019CZ	1			
	2018CZ	0.831**	1		
	2017CZ	0.591**	0.597**	1	
	BLUP	0.946**	0.938**	0.668**	1

FLW, flag leaf width; FLL, flag leaf length; AD, anthesis date; PH, plant height; SL, spike length; SNS, spikelet number per spike; SD, spike density; **Significance level at P = 0.01.

**TABLE 3:** Phenotypic correlations between seven yield-related traits with BLUP data in ‘AS985472’/‘Sumai 3’ (AS) population.

	**AD**	**FLW**	**FLL**	**PH**	**SL**	**SNS**	**SD**
AD	1						
FLW	–0.144	1					
FLL	0.269**	0.129	1				
PH	0.198	–0.047	0.143	1			
SL	0.446**	0.111	0.455**	0.466**	1		
SNS	0.385**	0.230**	0.404**	0.237**	0.668**	1	
SD	−0.251**	0.072	−0.229**	−0.409**	-0.724**	0.019	1

AD, anthesis date; FLW, flag leaf width; FLL, flag leaf length; PH, plant height; SL, spike length; SNS, spikelet number per spike; SD, spike density; **Significance level at P = 0.01.

### QTLs Analysis

**FIGURE 3:**
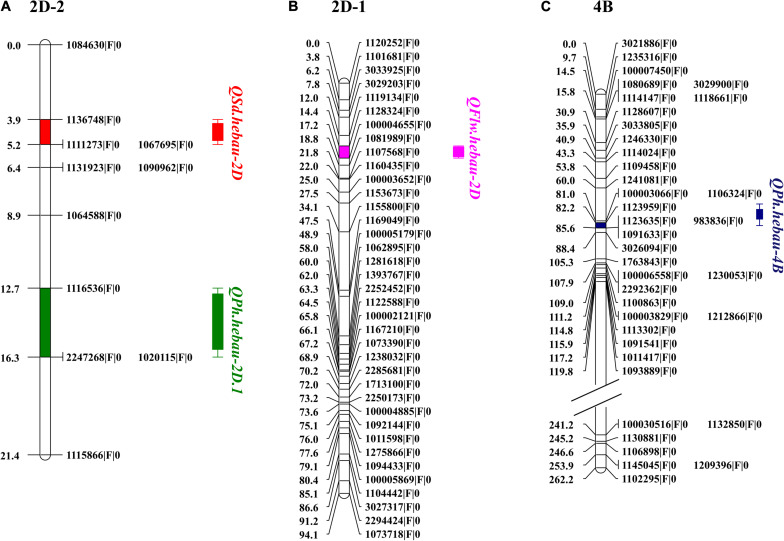
Distributions of QSd.hebau-2D, QPh.hebau-2D.1 **(A)**, QFlw.hebau-2D **(B)**, and QPh.hebau-4B **(C)** on the linkage map chromosome 2D and 4B, respectively.

QTLs conferring FLW, FLL, AD, PH, SL, SNS, and SD were detected in the AS population (Table 4). A total of two putative FLW QTLs (QFlw.hebau-2D and QFlw.hebau-3D) were identified on chromosomes 2D and 3D which individually explained 19.30 – 24.88% and 15.58% of the phenotypic variance, respectively; and the positive alleles of both were contributed by ‘Sumai 3’. Among them, QFlw.hebau-2D, a major stable QTL flanked by 1128324| F| 0 and 1081989| F| 0 (Table 4 and Figure 3), was detected in three environments and was confirmed by the BLUP dataset. For FLL, only a minor QTL (QFll.hebau-5A-1) was detected. With respect to AD, three QTLs with > 10% of the phenotypic variance were identified on chromosomes 2A and 2D, while they were detected in only one environment. For PH, six putative QTLs were mapped to chromosomes 2D, 4A, 4B and 6A. Two major and stable QTLs (QPh.hebau-2D.1, and QPh.hebau-4B) controlling PH accounted for the phenotypic variance up to 12.69 and 30.68%, and they were flanked by 1116536| F| 0 – 2247268| F| 0, and 1123959| F| 0 - 1123635| F| 0, respectively. ‘AS985472’ contributed the major alleles for increased PH at QPh.hebau-2D.1, and ‘Sumai 3’ contributed alleles for increased PH at QPh.hebau-4B. For SL, we detected eight loci, and two of the QTLs were co-localized with the major QTLs for SNS (QSns.hebau-4A) and for SD (QSd.hebau-6D.1). For SNS, seven QTLs with the 7.55 – 20.96% of the phenotypic variance were identified on chromosomes 2D, 3B, 4A, 5A, 5D, 7A, and 7B. Five QTLs for SD were mapped to chromosomes 2D, 3D, 4B, and 6D, of these, the major and stably expressed QTL QSd.hebau-2D accounted for 10.19–14.91% of the phenotypic variance and ‘Sumai 3’ contributed the major alleles at this locus.

**TABLE 4:** Quantitative trait loci (QTL) analysis for seven yield-related traits with single environment method and BLUP data.

**Trait**	**QTLs**	**Environments**	**Interval**	**Flanking maker**	**LOD**	**PVE (%)**	**Add**
FLW (cm)	QFlw.hebau-2D	2017CZ	14.39 – 17.20	1128324| F| 0 ∼ 100004655| F| 0	3.63	20.90	–0.11
		2019CZ	17.20 – 18.84	100004655| F| 0 ∼ 1081989| F| 0	5.07	24.88	–0.09
		2018CZ	17.20 - 18.84	100004655| F| 0 ∼ 1081989| F| 0	4.24	20.31	–0.09
		BLUP	17.20 – 18.84	100004655| F| 0 ∼ 1081989| F| 0	4.16	19.30	–0.08
	QFlw.hebau-3D	2017CZ	48.40 – 49.45	2323109| F| 0 ∼ 100005360| F| 0	2.65	15.58	–0.09
FLL (cm)	QFll.hebau-5A	BLUP	75.90 – 78.26	1136364| F| 0∼3028423| F| 0	6.54	0.67	0.62
PH (cm)	QPh.hebau-2D.1	2019CZ	12.68 – 16.32	1116536| F| 0∼2247268| F| 0	3.86	12.69	5.45
		2018CZ	12.68 – 16.32	1116536| F| 0∼2247268| F| 0	5.79	10.10	5.38
		BLUP	12.68 – 16.32	1116536| F| 0∼2247268| F| 0	8.25	11.30	5.36
	QPh.hebau-2D.2	2019CZ	7.82 – 12.03	3029203| F| 0∼1119134| F| 0	3.63	11.74	–5.40
	QPh.hebau-4A.1	BLUP	76.74 – 77.42	2290028| F| 0∼1121845| F| 0	7.10	9.41	–4.94
	QPh.hebau-4A.2	2018CZ	79.41 – 80.26	1372725| F| 0∼1115816| F| 0	7.38	13.26	–6.19
	QPh.hebau-4B	2019CZ	82.24 – 85.62	1123959| F| 0∼1123635| F| 0	6.84	24.74	–7.68
		2018CZ	82.24 – 85.62	1123959| F| 0∼1123635| F| 0	12.18	25.51	–8.67
		2017CZ	82.24 – 85.62	1123959| F| 0∼1123635| F| 0	4.82	30.68	–8.16
		BLUP	82.24 – 85.62	1123959| F| 0∼1123635| F| 0	17.10	29.85	–8.86
	QPh.hebau-6A	2018CZ	60.43 – 64.22	1124209| F| 0∼1283575| F| 0	7.50	13.52	6.25
		BLUP	60.43 – 64.22	1124209| F| 0∼1283575| F| 0	10.02	14.23	6.03
SL (cm)	QSl.hebau-2A	2017CZ	110.73 – 113.18	2254084| F| 0∼3026394| F| 0	2.84	13.64	–0.54
	QSl.hebau-2D	2019CZ	6.43 – 8.93	1090962| F| 0∼1064588| F| 0	3.33	10.83	0.45
	QSl.hebau-4A	2019CZ	90.26 – 91.09	1115627| F| 0∼100035209| F| 0	4.18	13.89	–0.51
	QSl.hebau-5A.1	2018CZ	20.01 – 21.26	3064643| F| 0∼3029299| F| 0	3.29	14.41	–0.46
	QSl.hebau-5A.2	2019CZ	76.83 – 89.00	1664450| F| 0∼1142113| F| 0	3.96	13.53	–0.51
	QSl.hebau-6D.1	2017CZ	9.46 – 13.31	100002192| F| 0∼1132651| F| 0	4.46	22.52	–0.70
	QSl.hebau-6D.2	2018CZ	15.80 – 16.88	100007946| F| 0∼1096139| F| 0	2.65	11.55	–0.41
	QSl.hebau-6D.3	BLUP	19.19 – 20.98	1138521| F| 0∼1127306| F| 0	3.35	12.59	–0.44
SNS	QSns.hebau-2D	2019CZ	3.76 – 6.23	1101681| F| 0∼3033925| F| 0	3.99	16.53	–0.66
		BLUP	3.76 – 6.23	1101681| F| 0∼3033925| F| 0	3.04	14.69	–0.56
	QSns.hebau-3B	20118CZ	112.75 – 113.74	3034270| F| 0∼1769222| F| 0	3.37	8.58	0.51
		2019CZ	112.75 – 113.74	3034270| F| 0∼1769222| F| 0	2.55	10.79	0.52
	QSns.hebau-4A	2017CZ	90.26 – 91.09	1115627| F| 0∼100035209| F| 0	3.41	22.96	–0.72
		2018CZ	90.26 – 91.09	1115627| F| 0∼100035209| F| 0	5.29	13.50	–0.63
	QSns.hebau-5A	2019CZ	65.32 – 73.19	2275311| F| 0∼1110394| F| 0	3.47	14.14	–0.59
	QSns.hebau-5D	2018CZ	0 – 3.55	2245326| F| 0∼1269099| F| 0	4.52	11.11	–0.57
	QSns.hebau-7A	2018CZ	208.95 – 212.16	1067518| F| 0∼1115252| F| 0	3.08	7.55	–0.47
	QSns.hebau-7B	2018CZ	96.84 – 97.69	1202000| F| 0∼1229729| F| 0	6.41	17.22	0.71
SD	QSd.hebau-2D	2019CZ	3.95 – 5.17	1136748| F| 0∼1111273| F| 0	2.81	10.19	–0.05
		2018CZ	3.95 – 5.17	1136748| F| 0∼1111273| F| 0	2.95	12.83	–0.05
		BLUP	3.95 – 5.17	1136748| F| 0∼1111273| F| 0	3.74	14.91	–0.05
	QSd.hebau-3D	2018CZ	33.84 – 46.08	100003200| F| 0∼1203665| F| 0	2.62	11.22	0.04
	QSd.hebau-4B	2019CZ	60 – 80.99	1241081| F| 0∼100003066| F| 0	2.77	12.36	0.06
	QSd.hebau-6D.1	BLUP	9.46 – 13.31	100002192| F| 0∼1132651| F| 0	3.67	14.58	0.05
	QSd.hebau-6D.2	2019CZ	14.63 – 15.80	100035082| F| 0∼100007946| F| 0	3.43	12.71	0.06
		2018CZ	14.63 – 15.80	100035082| F| 0∼100007946| F| 0	2.84	12.32	0.05
AD (d)	QAd.hebau-2A	2018CZ	20.56 – 21.68	100004846| F| 0∼2361439| F| 0	3.29	16.07	1.58
		BLUP	20.56 – 21.68	100004846| F| 0∼2361439| F| 0	2.81	11.07	0.88
	QAd.hebau-2D.1	2017CZ	0 – 3.95	1084630| F| 0∼1136748| F| 0	3.54	21.70	2.47
	QAd.hebau-2D.2	2019CZ	16.32 – 21.36	1020115| F| 0∼1115866| F| 0	3.76	18.59	1.59
		BLUP	16.32 – 21.36	1020115| F| 0∼1115866| F| 0	3.63	15.23	1.03

*QTL, quantitative trait loci; PVE, Phenotypic variance explained; LOD, logarithm of odds; Add, Additive effect of a QTL; positive values: alleles from ‘AS985472’ are increasing the trait scores; negative values: alleles from ‘Sumai 3’ are increasing the scores; FLW, flag leaf width; FLL, flag leaf length; AD, anthesis date; PH, plant height; SL, spike length; SNS, spikelet number spike; SD, spike density; CZ, Chongzhou; BLUP, best linear unbiased prediction.*

**FIGURE 4:**
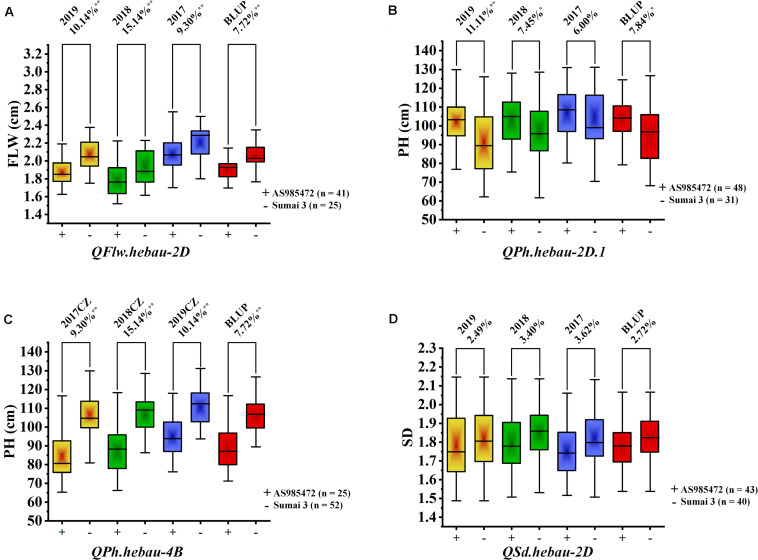
The effects of the QTL QFlw.hebau-2D on flag leaf width **(A)**, QPh.hebau-2D.1 on plant height **(B)**, QPh.hebau-4B on plant height **(C)** and QSd.hebau-2D on spike density **(D)**. Box plots represent RILs with and without major alleles which are grouped according to the flanking markers of the major QTLs. ‘+’ indicates the homozygous lines from ‘AS985472’ and ‘–’ indicates the homozygous lines from ‘Sumai 3’. **Significant at P = 0.01, *Significant at P = 0.05.

The 94 AS RILs were divided into two groups according to the genotypes of the two flanking markers for major loci QFlw.hebau-2D, QPh.hebau-2D.1, QPh.hebau-4B, and QSd.hebau-2D. Student’s t-test showed that RILs with the increased alleles from ‘Sumai 3’ significantly increased FLW in different environments as well as the BLUP dataset (P < 0.01, Figure 4A); and the lines with the positive alleles at QPh.hebau-2D from ‘AS985472’ were higher (P < 0.05, Figure 4B) than those from ‘Sumai 3’ in all environments except 2017CZ. In contrast, the lines with the increased alleles at QPh.hebau-4B from ‘Sumai 3’ were significantly (P < 0.01, Figure 4C) higher than those without major alleles. However, there were no differences between the lines with and without increasing alleles from *QSd.hebau-2D* (Figure 4D).

## Discussion

**FIGURE 5:**
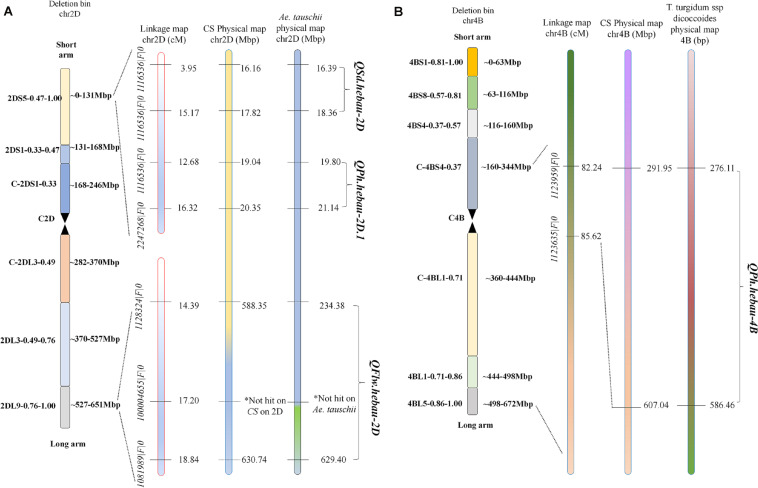
The maps of QFlw.hebau-2D/QPh.hebau-2D.1/QSd.hebau-2D **(A)** and QPh.hebau-4B **(B)**.

Many QTL identified in this study were closely located to the chromosome region of known QTL. For example, *QPh.hebau-2D.1* for PH in the present study were located at 19.04 – 20.35 Mbp on CS 2DS and 19.80 – 21.14 Mbp on *Ae. tauschii* 2DS (Figure 5A), whereas *QPh.hebau-4B* located within the interval of 291.95 – 607.04 Mbp on CS 4BL and 276.11 – 586.46 Mbp on wild emmer 4BL (Figure 5B). The location of *QPh.hebau-4B* (291.95 – 607.04 Mbp) is far from RhtB1 (30.861–30.863 Mbp). These two QTL were physically located at the similar or overlapped positions as those reported previously by Wu et al. (2010) and Zhai et al. (2016), respectively. It is interesting that the height reducing allele of *QPh.hebau-2D.1* came from the tall parent Sumai 3. This result suggested that genotypes with a relatively poorer performance on height may still carry the beneficial allele that can be used for genetic improvement of the trait. Similar results was obtained in previous study. It has been shown that susceptible parent contributed the resistance alleles to various wheat diseases (Poole et al., 2012; Ma et al., 2019b). In addition to height QTL, a major and stably expressed QTL conferring SD designated as *QSd.hebau-2D*, which was located at 16.16 – 17.82 Mbp on CS and 16.39 – 18.36 Mbp on *Ae. tauschii* 2DS (Figure 5A), was in the overlapped physical region as the QTL reported by Heidari et al. (2011). The stable expression of these height and SD QTL under multiple genetic backgrounds in different studies emphasized their value for further fine mapping studies.

Two putative QTLs for FLW were detected on chromosomes 2D and 3D including *QFlw.hebau-2D* and *QFlw.hebau-3D*. *QFlw.hebau-2D*, located in the interval of *1128324| F| 0* – *1081989| F| 0*, was the major and stable locus identified in the present study. For chromosome 2D, numerous putative loci for flag-related traits and yield-related traits in wheat were identified (Fan et al., 2015; Ma et al., 2019a, 2020). In the present study, *QFlw.hebau-2D* was located in a 4.45 cM interval and physically mapped between 588.35 and 630.74 Mbp on CS 2DL and 234.38 and 629.40 Mbp on *Ae. tauschii* 2DL (Figure 5A). Combined with the results from Ma et al. (2020), we reviewed the recently published articles related to FLW loci and compared the major FLW QTL detected in the present study with previous studies. Comparison analysis showed that it was overlapped with *qFlw-2D.2* (Fan et al., 2015) that was detected in an individual environment with minor effect. The large PVE (24.88) and LD (5.07) of *QFlw.hebau-2D* in our study suggested that value of this locus for FLW improvement.

Six hundred and eighty-nine putative candidate genes on CS genome were predicted at the interval of *QFlw.hebau-2D* (Supplementary Table S1). The results of the annotation indicated that several genes associated with plant growth and development. Additionally, there are numerous genes encoding the same protein. For example, auxin response factors, encoded by *TraesCS2D01G491200* and *TraesCS2D01G548900*, bind to TGTCTC auxin response elements in promoters of early auxin response genes (Tiwari et al., 2003), and thus regulating the development of plant tissues and organs. Twenty-two candidate genes including *TraesCS2D01G524300* encode an F-box protein which regulates floral development and the F-box protein TIR1 is an auxin receptor that mediates Aux/IAA degradation and auxin-regulated transcription (Dharmasiri et al., 2005). Flowering Locus T-like protein, encoded by *TraesCS2D01G538000* and *TraesCS2D01G538100*, is involved in the switch to flowering and thus aiding in grain set and dispersal.

MADS-box transcription factors are involved in various processes of plant growth and development (Ma et al., 2017). *TraesCS2D01G529700* encodes MADS-box transcription factor 8. Twelve genes encode SAUR-like auxin-responsive family proteins, which regulate cell expansion or division thus leading to leaf growth (Ren and Gray, 2015). These results showed that some of the putative candidate genes analyzed in the present study have key importance in understanding the genetic mechanism of flag leaf growth and development in wheat.

Significant genetic associations among seven yield-related traits were detected. SD was determined by the SNS divided by SL, thus SD was positively related with SNS and negatively related with SL, which was consistent with the phenotypic correlations in this study (Table 3). Significantly, several QTLs for SL with pleiotropic effects to SNS and SD that are the key components of grain yield in wheat were identified on chromosomes 4A and 6D, indicating that these traits may be controlled by the same locus. Major loci *QSd.hebau-2D* and *QPh.hebau-2D.1* were physically located at 16.16–17.82 Mbp and 19.04–20.35 Mbp, respectively, suggesting the intrinsic correlations may exist between SD and PH. Similarly, it is previously reported that various yield-related traits like biomass and SD (Kirigwi et al., 2007), SD and SL (Heidari et al., 2011), are controlled by the same QTLs or genes. The pleiotropic effects of these loci further indicated their potential value for further researches and application in wheat breeding programs.

## Data Availability Statement

All datasets presented in this study are included in the article/Supplementary Material.

## Author Contributions

JM and WZ designed the research. JJ, DL, and YQ performed the experiments. JJ and DL analyzed the data and wrote the manuscript. JM and JJ revised the manuscript. All authors read and approved the manuscript.

## Conflict of Interest

The authors declare that the research was conducted in the absence of any commercial or financial relationships that could be construed as a potential conflict of interest.

## Footnotes

1 https://urgi.versailles.inra.fr/download/iwgsc/

2 https://www.ncbi.nlm.nih.gov/assembly/GCA_002575655.1/#/def_asm_Primary_Assembly

3 https://www.dropbox.com/sh/3dm05grokhl0nbv/AAC3wvlYmAher8fY0srX3gX9a?dl=0%2

## Supplementary Material

TABLE S1 Predicated genes in the interval of the major QTL *QFlw.hebau-2D.*
